# Patient Directed Social Media Use Among Participants of Centering Pregnancy Groups

**DOI:** 10.21203/rs.3.rs-6872774/v1

**Published:** 2025-07-07

**Authors:** Alison M. El Ayadi, Nadia Diamond-Smith, Mia Schuman, Laura Weil

**Affiliations:** University of California San Francisco; University of California San Francisco; University of California San Francisco; University of California San Francisco

**Keywords:** group prenatal care/Centering Pregnancy^®^, antepartum care, postpartum care, health informatics, mental health, patient education

## Abstract

**Introduction:**

Social support in pregnancy and postpartum is important for optimizing maternal and infant health. Group prenatal care offers the opportunity for in-person social support yet does not extend into the postpartum period. Mobile social support models may further meet the needs of pregnant persons and partners during pregnancy and into the postpartum period. However, the use and utility of mobile social support for pregnant people and their partners in the context of group prenatal care and beyond has not been studied. Assessing Centering patients’ utilization of existing social media platforms can inform programmatic development.

**Methods:**

We conducted a retrospective cross-sectional study among recent participants of UCSF’s Centering Pregnancy^®^ program and their partners. Study participants were recruited through UCSF’s electronic health record system or direct email and partners were recruited through referral from participants. Online surveys sought to understand participant perspectives on mobile groups, educational and social support needs, and recommendations.

**Results:**

Participants gave birth between 2018–2021 (68%), were college-educated (97.3%), and regularly accessed social media (>75% across platforms). Most participants engaged in Centering Pregnancy^®^ online communications outside of formal activities (79% during pregnancy, 74% postpartum) for social support (78.1%) and knowledge sharing (65.2%). Most posted content monthly (54.6%) but read content more frequently (48.0% at least weekly). Communication frequency and topics changed with the COVID-19 pandemic. Respondents wanted more information on infant sleep (42.6%), maternal recovery/health (38.7%), breastfeeding/formula feeding (37.4%), newborn health and care (34.2%), and child development (27.1%). Social group engagement was higher for individuals reporting depressive or anxiety symptoms during pregnancy and postpartum.

**Discussion:**

The findings confirm the importance of social support, especially postpartum, for health, how mobile support groups can impact these outcomes, and needs and areas of improvement for group prenatal care. Integration of a social media support component to the evolving post-COVID Centering Pregnancy^®^ model may be an important addition to improve participating parent wellbeing.

## Introduction

Pregnancy and postpartum social support is associated with improved maternal physical and mental health, and improved birth outcomes in high-risk populations.^1–3^ However, perinatal social support interventions are largely neglected in current mainstream care models, particularly in the postpartum period. Women with low self-reported social support have been found to be at higher risk of depressive symptoms, low quality of life scores, and among certain subgroups, at higher risk of preterm birth and having low birth weight infants.^2,4,5^ Innovative, low cost approaches can ensure that women receive the emotional, instrumental, informational, and appraisal support needed during a stressful and often isolating time.^6^

Social media use in the pregnancy and postpartum period has come into focus as an opportunity for augmenting emotional and informational support during the COVID-19 pandemic and immediate post-pandemic landscape. Studies report the majority of recently pregnant patients use social media for information and advice pertaining to pregnancy and parenting (89%);^7–9^ in one study, respondents reported their *“friends from social media”* as key social support sources as a parent (83%).^7^ Many studies have developed social media interventions to address the support needs of pregnant and postpartum patients. A recent meta-analysis determined that social media can be effective in promoting healthy behaviors regarding gestational diabetes, weight control and maternal mental health,^10^ and a scoping review of social media perinatal health promotion interventions demonstrated significant improvements in breastfeeding and parenting confidence.^11^ These findings from both observational and interventional studies signifies that the provision of social support through social media platform is promising, particularly through a small group orientation.

Fostering social support is an explicit focus of group prenatal care models such as Centering Pregnancy^®^, women’s self-help groups, and others. Group prenatal care models offer prenatal care within a group setting, with time dedicated to clinical assessment, education, discussion and social interaction. The goal of these models is to provide efficient care and promote better birth outcomes, more provider and patient contact, patient learning and empowerment, self-care, support and friendship among group members. Each session includes facilitated educational activities and group discussion. Partners are also included in this model as a way of engaging and educating partners and increasing support for the birthing person. Typically, group sessions occur during the second and third trimesters of pregnancy, with some groups holding one final group meeting postpartum. However, these models do not generally extend further into the postpartum period. These models are gaining popularity in the U.S. due to their high acceptability and improved outcomes including: reduction in preterm birth and caesarean sections, decreased low birth weight, improved maternal weight gain, increased exclusive breastfeeding, postnatal contraceptive uptake and infant vaccination rates, and reduced postnatal depression.^12–21,22^ Women from minoritized racial or ethnic groups and/or disadvantaged communities have shown the most significant improvements in outcomes from group prenatal care.^23^

The University of California San Francisco (UCSF) Women’s Health Center has been a leader in expanding access to group prenatal care. One recent innovation is that some group prenatal care providers are recommending that their groups also form a mobile social support group to promote interaction outside of the formal, provider-facilitated meetings, including into the postpartum period when group prenatal care ceases. Anecdotally, we know that some groups have started communicating on WhatsApp and other forums, yet no known studies have explored the feasibility, acceptability, and impact of mobile social support groups on social support or described major content areas of these mobile social support groups among Centering Pregnancy participants. Understanding the role of mobile social support in addition to group prenatal care is important for the continued refinement of this model and identifying what additional support and information pregnant persons and their partners seek outside of group care can help providers better serve patients throughout pregnancy and postpartum. Mobile social and education support became of increased interest during the recent COVID-19 pandemic where care moved online as much as possible, and there was an increased need to provide high quality care through virtual models.

We sought to understand the use of social media as adjunct to the group care model among UCSF’s Centering Pregnancy^®^ participants (birthing people and their partners) both during and after pregnancy, focusing on group formation, participation and dynamics, main topics of discussion, perceived value added and drawbacks to the adjunct social media use, and mental health correlates.

## Methods

We conducted a retrospective cross-sectional study among recent participants of UCSF’s Centering Pregnancy^®^ program. Centering Pregnancy^®^ has been an integral part of prenatal care provision at UCSF since 1999. Approximately 150–200 women participate in Centering Pregnancy^®^ annually. Traditionally, UCSF has launched 1–2 groups each month, led by a faculty midwife or physician. Many groups involve obstetrics and gynecology residents. Groups are opt-in and there is considerable demand in the community with a waitlist for participation. Patients are enrolled after the initial obstetrics intake appointment and include both low and high-risk patients, some with co-management by a high-risk obstetrician.

Potential participants were recruited via UCSF’s electronic health record (EHR) system and via direct program email. Individuals meeting the study inclusion criteria of having attended at least one group prenatal care visit in the prior five years and giving birth to a live infant were sent a message through the patient-provider messaging system which included a summary description of the research aims, what participating in the survey entailed, how confidentiality would be ensured, and potential risks or benefits. Individuals who responded that they were interested in learning more about the study were connected to the research team who shared further details regarding the study procedures and a link for online participation. Participants were asked to confirm informed consent by checking a box before being allowed to move into the survey. We did not collect names or other identifying information for research purposes; however, we did separately collect email addresses for the sole purpose of distributing a participation incentive of a $10 Amazon.com gift card. In recruitment emails and at the end of the patient survey, we shared a link for participants to invite their partners to participate in a similar online survey.

The participant survey included questions on sociodemographic characteristics (age, parity, educational attainment, race/ethnicity, and general social media use) to describe our sample. We also collected information on sources of social support (technological, in-person, etc.), estimated frequency of engagement, and changes in social support engagement across the pregnancy and postpartum continuum. We collected details on social media group participation, information on the mobile platform they used, and their perspectives on the social media group and social support mechanisms available more broadly, what benefits and challenges it presented, how valuable they felt it was, their views on its impact on support, and how it could have been improved. We asked about women’s perspectives on how the various sources of social support impacted their mental health during the pregnancy and postpartum period, and their experiences of depressive and anxiety symptoms. Finally, we asked what additional information or support participants would have liked to have at these stages, and what their recommendations would be for a more formal social media group within the context of UCSF’s Centering Pregnancy^®^ program.

Over the period October 22 – December 31, 2020, a total of 731 individuals were identified as eligible for participation and sent the initial recruitment email within UCSF’s EHR messaging system; 106 responded that they were interested in the study, and 81 women completed the online survey. At survey recruitment and completion, we indicated an additional interest in recruitment of partners and shared a link to a partner survey. Twelve partners completed the online survey. A second round of recruitment was conducted by email due to the limited number of respondents to the MyChart request over July 15 – August 16, 2022. The email recruitment went out to all 234 pregnant people who participated in virtual Centering pregnancy, an adaptation of traditional, in-person Centering necessitated by the covid pandemic. The virtual groups were held from 2020 through 2023 when a hybrid model was established. The 74 respondents were similar in demographic characteristics to the first round of recruitment and reflective of the larger Centering program at UCSF. The second sample included patients who were currently pregnant and actively receiving group care. Partners were recruited using the same strategy.

Reporting of results focuses on data shared by the birthing person; however, we do present partner data where available, though the sample size is considerably smaller. Data analyses were largely descriptive in nature and missing data are noted. We characterized survey participants’ socio-demographic characteristics, their engagement in adjunct social media group, and their emotional and informational support and needs through presenting ns and proportions. We estimated the relationship between frequency of social media group engagement (reading and posting) and birthing person report of depression and anxiety (operationalized as always or often vs. sometimes, rarely or never) during pregnancy and postpartum through logistic regression analysis. We compared report of depression and anxiety during pregnancy through postpartum using Stuart-Maxwell’s test.

Study procedures and data collection instruments were approved by the UCSF Human Research Protection Program IRB#:20–30838. All participants provided online confirmation of informed consent. This research article was written in accordance with the STROBE guidelines for cross-sectional studies.

## Results

### Survey participant characteristics

Our 155 study participants ranged in age from 27 to 46, with most between age 35–39 (49.7%; Table 1). Most gave birth between 2018 through 2021 (67.7%). Most participants in the Centering Pregnancy^®^ program were experiencing their first pregnancy (63.9%). Nearly all participants were college-educated (97.3%), with 65.8% having completed a graduate degree. Most identified as white (60.0%). Participants were well versed in online communication and regularly accessed social media and other electronic communication platforms weekly including Facebook or Instagram (75.5%), email (74.2%), WhatsApp or other text applications (74.2%). Participation in Centering Pregnancy^®^ in-person meetings was high, with 93.5% reporting having participated in all or most of the group meetings (not shown).

Twenty-two partners participated in the survey (Table 1). Most were aged 35–39 (61.9%), college educated (95.3% bachelors or graduate degree), and identified as white (47.6%) or Asian (38.1%). Most (93.5%) reported participating in all or most of the Centering Pregnancy^®^ meetings (not shown), many for births occurring in 2020 (33.3%) or 2021 (19.0%).

### Participation in social media groups adjunct to Centering Pregnancy^®^

Most survey participants reported that their Centering Pregnancy^®^ groups communicated outside of the in-person group meetings (online) during pregnancy (79.1%) and postpartum (74.2%), and they participated in these communications (80.0% and 76.4%, respectively; Table 2). The most common platforms used were WhatsApp (62.9%) and email (43.6%), followed by Slack (13.6%) and Facebook (12.1%). Most social media groups included both pregnant/birthing people and their partners (58.7%) in a combined group. Social media group participation was good, with two-thirds of participants saying that all or most of their Centering Pregnancy^®^ group participated (67.6%). Among those who reported participating in their Centering Pregnancy^®^ group’s social media group during pregnancy, most posted content monthly (54.6%) but read content more frequently (48.0% at least weekly). More frequent participation was reported postpartum, with over half posting content at least weekly (54.5%) and about two-thirds reading content at least several times per week (63.3%). Participants reported engaging in the social media group for social support (78.1%) and knowledge sharing (65.2%). Most pregnant participants engaged in other support groups online (59.4%) or in-person (26.5%).

Partners reported engaging in their Centering Pregnancy^®^ group’s online communications during pregnancy (42.1%) and postpartum (42.1%), largely reporting they participated for knowledge sharing (45.5%), and social support (36.4%; Table 2). Most partners reported no other social media group (77.3%). Self-report by partner respondents were somewhat different than Centering Pregnancy participant report of partner engagement which suggested that 78.6% of partners engaged in the in-person group, 18.1% reported in social media group during pregnancy and 17.0% postpartum (not shown).

### In-person engagement with Centering Pregnancy^®^ group outside of scheduled group meetings

Unfacilitated in-person meetings were reported by 29.0% during pregnancy and 58.1% postpartum (Table 3). In-person meetings largely occurred every few months (60.0%) or monthly (11.1%) during pregnancy and every few months (45.6%), monthly (17.8%) or weekly (12.2%) postpartum. Many survey participants reported that they continued to interact with their Centering Pregnancy^®^ group in-person for a year or more after giving birth (39.4%), and (20.2%) reported they were still meeting. No relationship was identified between engagement in posting or reading and in-person meetings (not shown).

#### Impact of COVID-19 on Centering Pregnancy^®^ social media group

Given the timing of our study, we also asked whether there was any change to the social media group interaction due to COVID-19 and found diverse experiences (not shown). Among participants with ongoing groups, 41.9% reported no change, 24.7% reported more frequent interactions, and 33.3% reported less frequent interactions. One-third of ongoing group participants indicated that there was a change to the content of group interaction since COVID-19 (34.8%). Content shifts described included managing COVID-related risks, pandemic-related stress and mental health, managing working from home and childcare during the pandemic, and ideas for home-based activities. Several people shared that meet-ups occurred less frequently or not at all due to social distancing, and many people moved away. Sentiments are featured in the below participant narratives:

We talk about how to manage risks for our families, the decision to go back to daycare, etc. many have also since moved away, so the WhatsApp group has taken on new significance in that it’s really the only contact we’ll still have with each other on a regular basis. Before Covid we’d get together almost monthly with the babies.

Now that people are varied in the activities they engage in, some are more hunkered down than others, there isn’t as much conversation. Back when we were first newborn moms, we were on a journey together and could also get together more frequently, so we would be chatting each other at all hours of the day for advice, feedback or just venting.

All of us delivered shortly before social distancing began, so we were never able to meet up in person postpartum. We spoke more about navigating postpartum without additional help from family, etc.

### Topics most discussed and most addition information desired

The most commonly discussed topics (mentioned by 40% or more of birthing respondents) were birth experience, general pregnancy questions, common discomforts of pregnancy, and infant sleep (Table 4). Partners mentioned products and services, general pregnancy questions, breastfeeding/infant feeding, infant sleep, child development, and introduction of solids foods most commonly (mentioned by more than 40%). Overall, partners listed fewer discussion topics than birthing people.

The most frequently reported topics respondents wanted more information on were infant sleep (42.6%), maternal recovery/health (38.7%), breastfeeding/formula feeding (37.4%), newborn health and care (34.2%), and child development (27.1%; Table 4). Partners had fewer topics that they desired more information on, but the most common ones were infant sleep (18.2%), birth experience (13.6%) and partner relationship (13.6%).

### Emotional and informational support needs and mental health during pregnancy and postpartum

Centering participants largely reported that their emotional (86.5%) and informational (91.0%) support needs were very or somewhat well met during pregnancy; lower proportions reported these needs were met postpartum, at 63.3% reporting emotional and 69.7% informational needs met (Table 5). In corollary, endorsement of depressive and anxiety symptoms increased from pregnancy to postpartum, with 5.8% reporting depressive symptoms always or often antenatally compared to 18.6% postpartum (p < 0.002), and 9.0% reporting anxiety symptoms always or often antenatally compared to 22.1% postpartum (P = 0.009). Depressive symptoms during pregnancy were significantly associated with frequency of engaging with the social media group (reading content; Table 6); each increase in engagement frequency category was associated with a 2.32-fold increased odds of reporting depressive symptoms always or often during pregnancy (95% CI 1.17–4.61). Each increase in engagement frequency category was associated with increased odds of postpartum anxiety, including a 1.60-fold increased odds of reporting anxiety always or often postpartum (95% CI 1.10–2.34) associated with reading content and a 1.51-fold increased odds (95% CI 1.51–2.27) associated with posting content.

Emotional and information support was lower for partners and followed similar trends to participants reporting greater unmet need postpartum (Table 5): 61.9% reported emotional and 81.0% informational needs met during pregnancy compared to 47.4% of emotional and 63.1% of informational needs met postpartum. Report of depressive and anxiety symptoms increased from pregnancy to postpartum for partners as well: 9.5% prenatal and 15.8% postpartum endorsed depressive symptoms always or often whereas 19.0% prenatal and 27.3% postpartum endorsed anxiety symptoms always or often. These differences were not identified as statistically significant.

## Discussion

There was a clear increase in participation in social media groups for both patients and their partners in the postpartum period. Many of the questions related to infant care and feeding were actively discussed and still patients desired more information on these topics. This presents an opportunity for numerous interventions including resource packages that can be readily accessed on social media groups, facilitated postpartum support groups and in person postpartum group sessions. More generally, these findings suggest that birthing people and their partners want resources in the postpartum period, highlight the need to find additional and alternative ways of providing information and social support to people in this time period, which is often neglected.

Though the sample of partners responding was quite small, there is some interesting information to be gleaned.^24^ Partners had significantly more interest and activity in the social media group in the postpartum period. They were less likely to be involved in other social media groups and more likely to participate to engage with “trusted friends”. This presents an interesting opportunity for partner engagement in an often difficult to reach population. Past studies have also found that partners desired social support in the postpartum period around postpartum depression, which can afflict partners as well as the birthing person.^24^ Targeting social media groups for partners in the postpartum period may be an effective way to offer support and provide education.

The question of facilitation of social media groups was interestingly divided into three equal opinions: desire for facilitation, desire for no facilitation and unsure. This suggests that program developers may want to create a hybrid model of facilitation and independent social media participation. It is also possible that different options for facilitation approaches should be offered to participants, and there may be no one size fits all model.

Although conceptualized before the COVID-19 pandemic, this study was timely in that we were able to gather insights into people’s experiences of social media support and information groups during COVID-19. Our findings have implications given COVID-19 and the shift to more virtual care provision in the pandemic and into the future. Specifically, these types of resources (social media interventions) were highly valued and desired and have applicability beyond only the provision of information—they can also provide social support in a time where mental health is of particular importance and where engaging with others is more challenging. Recent studies have shown that COVID-19 led to increases in adverse postpartum mental health outcomes and that loneliness was a key contributing factor.^25^

This study has several strengths, including its ability to study a new practice among postpartum individuals, who have traditionally been neglected by the health care system, at a time with mobile and online resources are especially important given COVID-19. However, it did have several limitations. First, recruitment was relatively low and likely selective because of the difficulty of reaching patients through the MyChart portal. The majority of eligible participants never opened the MyChart message and were therefore unaware of the study; we conclude that participants are skewed to those individuals more actively engaged in health communication via this portal. Relatedly, partner recruitment was limited and relied on the patient to forward the recruitment link; we do not know whether low partner participation was due to lack of birthing individuals forwarding the link or low partner acceptance. Our findings are relevant to COVID-19 yet the fact that the data was collected during the pandemic could have led to biases in who completed the survey or their feelings about social media/online interactions. Our sample was reflective of birthing individuals who participate in Centering Pregnancy^®^ at UCSF more broadly, skewing to more educated, mostly white, and mostly first births, suggesting that more research in this area would be helpful to understand the generalizability of our findings within the broader US birthing population.

We identified demonstrated interest in some kind of postpartum online support. Given that it requires very little provider time or effort to recommend a patient organized, unfacilitated support group, it may serve the needs of patients to encourage this practice and be cost effective. Providers can also maintain a list of facilitated social media support groups that are local, issue or population specific to offer their patients.

The association between frequent use of the support group and depressive symptoms merits further exploration due to the significant associations identified. While directionality could not be determined from our study, further research should seek to understand if the use of social media by pregnant and postpartum patients is negatively affecting mood, or if the patients more prone to anxiety and depression are more likely to use the social media groups. These findings in particular have implications for the development of future online programs as the pandemic wanes and social media becomes a more integral part of communication worldwide.

## Conclusions

As the post-COVID Centering Pregnancy^®^ model evolves, attention should be paid to integrating a social media support component to the group that can be sustained into the postpartum period. More research is needed to develop an adjunct social support model for Centering Pregnancy^®^ groups, but the interest is clearly demonstrated.

## Figures and Tables

**Table 1:** Sociodemographic Characteristics of UCSF Centering Pregnancy^®^ Participants and Partners Engaging in Social Media Survey

	Participant (n = 155)		Partner (n = 22)	
	n	%	n	%
Age Group				
<30	5	3.2	0	0.0
30–34	51	32.9	5	23.8
35–39	77	49.7	13	61.9
40+	22	14.2	3	14.3
Year Gave Birth				
2016	9	5.8	1	4.8
2017	10	6.5	2	9.5
2018	29	18.7	1	4.8
2019	22	14.2	2	9.5
2020	20	12.9	7	33.3
2021	34	21.9	4	19.0
2022	23	14.8	2	9.5
Haven’t given birth	8	5.2	2	9.5
Number of times pregnant	(n = 153)			
1	99	64.7		
2–3	51	33.3		
4 or more	3	2.0		
Pregnancy participated in Group ANC				
First	99	63.9		
Second	56	36.1		
Educational attainment				
Some college	4	2.6	1	4.8
Bachelor’s degree	49	31.6	9	42.9
Graduate degree	102	65.8	11	52.4
Race/ethnicity	(n = 150)			
White	90	60.0	10	47.6
Asian	32	21.3	8	38.1
Multiple	13	8.7	1	4.8
Hispanic	9	6.0	1	4.8
Black	3	2.0	0	0.0
Other	3	2.0	1	4.8
Social media platforms used on weekly basis				
Emails	115	74.2	17	77.3
Facebook or instagram	117	75.5	10	45.5
Slack	51	32.9	10	45.5
Zoom or skype	73	47.1	12	54.5
WhatsApp or other texting	115	74.2	18	81.8
Other	8	5.2	3	13.6

**Table 2:** Adjunct Social Media Group Participation among UCSF Centering Pregnancy^®^ Participants and Partners Engaging in Social Media Survey

	Participant (n = 155)		Partner (n = 22)	
	n	%	n	%
Group communicated online				
No	8	5.2	1	4.5
During pregnancy	123	79.4	13	59.1
During postpartum	115	74.2	16	72.7
Don’t know	9	5.8	1	4.5
Participated in group’s online communications	(n = 140)		(n = 19)	
No^a^	7	5.0	8	42.1
During pregnancy	112	80.0	8	42.1
During postpartum	107	76.4	8	42.1
Platform used for communication	(n = 140)			
Email	61	43.6	4	21.1
Facebook	17	12.1	3	15.8
Slack	19	13.6	2	10.5
Zoom	12	8.6	1	5.3
WhatsApp	88	62.9	5	26.3
Google	2	1.4	0	0.0
Group target	(n = 143)			
All group participants	84	58.7	11	52.4
Pregnant people only	59	41.3	10	47.6
Proportion of group that participated in online group	(n = 142)			
All	23	16.2	1	5.3
Most	73	51.4	8	42.1
Some	36	25.4	7	36.8
Few	8	5.6	1	5.3
Do not remember	2	1.4	2	10.5
How often posted content during pregnancy	(n = 97)		(n = 7)	
Monthly	53	54.6	4	57.1
Weekly	21	21.6	3	42.9
Several times per week	22	22.7	0	0.0
Daily	1	1	0	0.0
How often read content during pregnancy	(n = 102)		(n = 8)	
Monthly	29	28.4	2	25.0
Weekly	24	23.5	2	25.0
Several times per week	26	25.5	1	12.5
Daily	15	14.7	3	37.5
Several times per day	8	7.8	0	0.0
How often posted content postpartum	(n = 101)		(n = 7)	
Monthly	46	45.5	3	42.9
Weekly	26	25.7	3	42.9
Several times per week	20	19.8	1	14.3
Daily	4	4	0	0.0
Several times a day	5	5		
How often read content postpartum	(n = 103)		(n = 7)	
Monthly	26	25.2	2	28.6
Weekly	17	16.5	3	42.9
Several times per week	28	27.2	2	28.6
Daily	17	16.5	0	0.0
Several times per day	15	14.6	0	0.0
How long after delivery continued to interact with group online	(n = 123)		(n = 14)	
Still meeting	24	19.5	0	0.0
4–6 weeks	18	14.6	5	35.7
6–12 weeks	6	4.9	1	7.1
3–6 months	6	4.9	1	7.1
6–12 months	21	17.1	1	7.1
12 + months	48	39	6	42.9
Reason participated in group				
Social support	121	78.1	8	36.4
Knowledge sharing	101	65.2	10	45.5
Trusted friends	27	17.4	5	22.7
Other	9	5.8	0	0.0
Would you have liked to have health provider join social media group			(n = 11)	
No	38	38.8	1	9.1
Yes	41	31.1	3	27.3
Unsure	53	40.2	7	63.6
Social media group still active			(n = 19)	
No	38	27.0	2	10.5
Yes	91	64.5	10	52.6
Don’t know	12	8.5	7	36.8
Participated in other online social media/in-person support groups				
No	41	26.5	17	77.3
Social media	92	59.4	4	18.2
In-person	41	26.5	0	0.0

Notes:

a Reasons for non-participation in group’s online communications were reported for CP Participants as other (n = 4), not enough time (n = 2), not comfortable with social media (n = 1), not comfortable sharing (n = 1), and no access to platform (n = 1). Partners reported other (n = 5), did not need additional support (n = 2), did not need additional information (n = 2), not comfortable with social media (n = 1), not enough time (n = 1), and no access to platform (n = 1).

**Table 3:** In-person engagement with Centering Pregnancy^®^ group among UCSF Centering Pregnancy^®^ Participants and Partners Engaging in Social Media Survey

	Participant (n = 155)		Partner (n = 22)	
	n	%	n	%
Group met in person outside of regular sessions	(n = 155)			
No	57	36.8	9	40.9
Yes, in pregnancy	45	29.0	4	18.2
Yes, postpartum	90	58.1	12	54.6
Frequency of in-person meetings during pregnancy	(n = 45)		(N = 4)	
Every few months	27	60.0	0	0.0
Monthly	5	11.1	3	75.0
Weekly	3	6.7	0	0.0
Several times per week	1	2.2		
Other	9	20.0	1	25.0
Frequency of in-person meetings postpartum	(n = 90)		(n = 12)	
Every few months	41	45.6	6	50.0
Monthly	16	17.8	3	25.0
Weekly	11	12.2	0	0.0
Several times a week	2	2.2	0	0.0
Other	20	22.2	3	25.0
How long after delivery continued to interact with group in-person	(n = 104)		(n = 12)	
Still meeting	21	20.2	0	0.0
4–6 weeks	15	14.4	2	16.7
6–12 weeks	6	5.8	1	8.3
3–6 months	8	7.7	2	16.7
6–12 months	13	12.5	1	8.9
12 + months	41	39.4	6	50.0

**Table 4:** Topics Discussed and Discussion Needs during Adjunct Social Media Group Participation for UCSF Centering Pregnancy^®^ Participants and Partners Engaging in Social Media Survey

	Discussed		Wanted More Discussion	
	n	%	n	%
***Centering Pregnancy^®^ Participants (N = 155)***				
Birth experience	104	67.1	32	20.6
General pregnancy questions	96	61.9	11	7.1
Common discomforts of pregnancy	84	54.2	16	10.3
Infant sleep	66	42.6	66	42.6
Maternal recovery/health	60	38.7	60	38.7
Pregnancy complications	59	38.1	24	15.5
Breastfeeding/formula feeding	58	37.4	58	37.4
Newborn health and care (bathing, skin care, colic, allergies, vaccinations)	53	34.2	53	34.2
Child development	42	27.1	42	27.1
Maternal mood	35	22.6	35	22.6
Introduction of solid food	32	20.6	32	20.6
Partner relationship	31	20	31	20
Return to sex, family planning, sex drive	29	18.7	29	18.7
Products and services	23	14.8	23	14.8
Other	8	5.2	8	5.2
**Partners (N = 22)**				
Products and services	10	45.5	0	0.0
General pregnancy questions	9	40.9	2	9.1
Breastfeeding/formula feeding	9	40.9	1	4.5
Infant sleep	9	40.9	4	18.2
Child development	9	40.9	2	9.1
Introduction of solid food	9	40.9	2	9.1
Birth experience	8	36.4	3	13.6
Maternal recovery/health	8	36.4	2	9.1
Newborn health and care (bathing, skin care, colic, allergies, vaccinations)	8	36.4	2	9.1
Common discomforts of pregnancy	7	31.8	1	4.5
Pregnancy complications	7	31.8	0	0.0
Maternal mood	5	22.7	1	4.5
Partner relationship	3	13.6	3	13.6
Return to sex, family planning, sex drive	2	9.1	1	4.5
Other	2	9.1	0	0.0

Notes: Newborn health and care^a^ (bathing, skin care, colic, allergies, vaccinations)

**Table 5:** Emotional and informational support needs and depressive and anxiety symptoms, during pregnancy and postpartum among UCSF Centering Pregnancy^®^ Participants and Partners Engaging in Social Media Survey

	Centering Participant (n = 155)				Partner (n = 12)			
	Pregnancy		Postpartum		Pregnancy		Postpartum	
	n	%	n	%	n	%	n	%
How well were your emotional support needs met?			(n = 142)					
Very well	70	45.2	35	24.6	6	28.6	5	26.3
Somewhat well	64	41.3	55	38.7	7	33.3	4	21.1
Neutral	13	8.4	23	16.2	7	33.3	5	26.3
Not very well	5	3.2	21	14.8	0	0.0	4	21.1
Poorly	3	1.9	8	5.6	1	4.8	1	5.3
How well were your informational support needs met?			(n = 142)					
Very well	92	59.4	46	32.4	6	28.6	2	10.5
Somewhat well	49	31.6	53	37.3	11	52.4	10	52.6
Neutral	7	4.5	20	14.1	4	19.0	6	31.6
Not very well	6	3.9	19	13.4	0	0.0	1	5.3
Poorly	1	0.6	4	2.8	0	0.0	0	0.0
Frequency of depressive symptoms			(n = 140)					
Always	0	0	4	2.9	0	0.0	0	0.0
Often	9	5.8	22	15.7	2	9.5	3	15.8
Sometimes	32	20.6	48	34.3	6	28.6	5	26.3
Rarely	65	41.9	52	37.1	10	47.6	9	47.4
Never	49	31.6	14	10	3	14.3	2	10.5
Frequency of anxiety symptoms	(n = 154)		(n = 140)					
Always	1	0.6	7	5	0	0.0	0	0.0
Often	13	8.4	24	17.1	4	19.0	5	26.3
Sometimes	39	25.3	38	27.1	4	19.0	3	15.8
Rarely	54	35.1	51	36.4	6	28.6	7	36.8
Never	47	30.5	20	14.3	7	33.3	4	21.1
Mental health diagnoses								
Depression	9	6.2	17	11.3	0	0.0	1	4.8
Anxiety	12	7.7	30	19.4	0	0.0	2	9.1

**Table 6:** Emotional and informational support needs and depressive and anxiety symptoms, during pregnancy and postpartum among UCSF Centering Pregnancy^®^ Participants

	Pregnancy				Postpartum			
	Depressive symptoms		Anxiety		Depressive symptoms		Anxiety	
	OR (95% CI)	P	OR (95% CI)	P	OR (95% CI)	P	OR (95% CI)	P
Frequency ***read*** social media group content	2.32 (1.17–4.61)	0.016	1.09 (0.62–1.92)	0.77	1.29 (0.89–1.86)	0.181	1.60 (1.10–2.34)	0.015
Frequency ***posted*** social media group content	1.51 (0.65–3.52)	0.342	0.72 (0.28–1.85)	0.49	1.03 (0.66–1.60)	0.897	1.51 (1.01–2.27)	0.046

Notes: Depressive symptoms and anxiety operationalized as always or often versus sometimes, rarely or never (reference).

## Data Availability

Data are accessible upon reasonable request by the corresponding author.
